# Comprehensive performance evaluation of a high-throughput automated system for pathogen nucleic acid detection in clinical settings

**DOI:** 10.3389/fmicb.2025.1609142

**Published:** 2025-06-16

**Authors:** Rongqi Lu, Rui Zhang, Yali Liu

**Affiliations:** ^1^Department of Laboratory Medicine, Peking Union Medical College Hospital, Chinese Academy of Medical Sciences, Beijing, China; ^2^Graduate School, Peking Union Medical College, Chinese Academy of Medical Sciences, Beijing, China

**Keywords:** clinical detection of pathogen nucleic acids, automation, high-throughput, performance verification, RT-PCR

## Abstract

**Objective:**

This study evaluates the clinical performance of a high-throughput automated molecular detection system and proposes a comprehensive and standardized performance validation framework to address gaps in existing methodologies and provide a robust reference for future evaluations.

**Methods:**

Performance was validated for EBV DNA, HCMV DNA, and RSV RNA using clinical samples at various concentrations, along with WHO and national reference standards. The validation included concordance rate, accuracy, linearity, precision, limit of detection, interference testing, cross-reactivity, and carryover contamination.

**Results:**

The positive, negative, and overall concordance rates for EBV DNA, HCMV DNA, and RSV RNA were all 100%. Both intra-assay and inter-assay precision showed coefficients of variation (CV) below 5%. The linear correlation coefficient (| r|) for EBV DNA and HCMV DNA was ≥ 0.98, demonstrating excellent linearity. The limits of detection (LoD) were 10 IU/mL for EBV DNA and HCMV DNA, and 200 copies/mL for RSV RNA. Both interference and cross-reactivity assessments met the CLSI EP07 standards, and no carryover contamination was observed.

**Conclusion:**

The system demonstrated excellent performance in terms of concordance, accuracy, precision, linearity, interference testing, and cross-reactivity. It is highly suited for large-scale pathogen screening and routine nucleic acid testing in clinical laboratories, both for qualitative and quantitative analyses. Additionally, this study introduces a comprehensive and standardized performance validation framework that addresses critical gaps in existing methodologies, offering a robust foundation for the rigorous evaluation of diagnostic systems and serving as a valuable reference for future research.

## Introduction

Since Mullis et al. proposed the classical Polymerase Chain Reaction (PCR) (Mullis et al., 1986), PCR technology has undergone continuous development, now encompassing several formats such as traditional PCR, quantitative real-time PCR (qPCR), and digital PCR (dPCR) (Zhu et al., 2020). During the COVID-19 pandemic, PCR technology has become the gold standard for detecting viral infections due to its high specificity, high sensitivity, and ability to rapidly and accurately identify viral nucleic acids (Trinh et al., 2023; Gupta et al., 2021). However, current PCR experiments often depend on multiple separate laboratory areas, involve complex operational procedures (Wee et al., 2020; Tan et al., 2022), and are prone to inaccurate results due to factors such as aerosol contamination, improper handling, cross-contamination between specimens, and amplicon contamination (Mwangi et al., 2022; Huggett et al., 2020; Zhou et al., 2020). These issues limit the widespread application of PCR technology in high-throughput settings. The experience gained from large-scale nucleic acid testing during the COVID-19 pandemic suggests that the future development of PCR technology will focus more on automation, and high-throughput processing (Safiabadi Tali et al., 2021). By integrating different PCR sections and developing integrated high-throughput nucleic acid analysis systems, it is possible not only to significantly improve the efficiency of PCR testing but also to reduce the need for manual labor, minimize human error, and lower the risk of laboratory contamination, ultimately achieving the goal of shortening turnaround time (TAT) for reports.

To address the challenges commonly encountered in PCR workflows, the high-throughput nucleic acid detection system evaluated in this study integrates all critical functions—including sample preprocessing, nucleic acid extraction, PCR setup, and amplification detection—into a fully automated, closed-loop platform. It incorporates advanced biosafety mechanisms such as physical partitioning, dual isolation doors, gradient negative pressure control, high-efficiency particulate air (HEPA) filtration, and ultraviolet (UV) disinfection, ensuring contamination-free operation even under continuous high-throughput conditions. In addition, its flexible configuration supports both qualitative and quantitative testing across a wide range of specimen types. By adopting this streamlined “sample in, result out” approach, the system significantly enhances operational efficiency, minimizes manual intervention, and reduces the risk of contamination in routine diagnostics, thereby meeting the diverse needs of clinical laboratories and public health settings.

Despite advances in automation, many published evaluations of molecular detection platforms continue to rely on limited performance indicators such as sensitivity, specificity, or precision alone (Tang et al., 2024; Nörz et al., 2021; Hildenbrand et al., 2018). This narrow approach often lacks the breadth needed to support clinical implementation or comparison across systems. To address this, our study adopts a comprehensive validation strategy based on protocols from the Clinical and Laboratory Standards Institute (CLSI), including EP05, EP06, EP07, EP09, EP12, EP17, and EP47. These guidelines are widely recognized by ISO 15189 and CAP-accredited laboratories worldwide for their methodological rigor and clinical relevance (Pum, 2019a; Pum, 2019b; Adeli et al., 2017). In China, CLSI protocols are routinely used as standard references in ISO 15189–certified institutions, including our own laboratory, which is accredited by both ISO 15189 and CAP.

Building upon prior evaluation efforts, this study aims to provide a systematic and multidimensional performance assessment of a high-throughput nucleic acid detection system. By simultaneously evaluating quantitative detection for EBV and HCMV and qualitative detection for RSV, the study offers an integrated framework that reflects both clinical diagnostic practice and regulatory expectations. The results are intended to serve as a reference model for future assay validations in similar high-throughput clinical settings.

## Materials and methods

### Sample and reference material sources

A total of 120 residual plasma samples from clinical testing for EBV and HCMV at the Department of Laboratory Medicine, Peking Union Medical College Hospital were used, with concentrations ranging from 3 × 10 IU/mL to 5 × 10^7^ IU/mL (82 positive and 38 negative cases). Additionally, 121 residual oropharyngeal swab samples were used for RSV testing (90 positive and 31 negative cases). Reference materials included the 1st WHO International Standard for EBV (NIBSC code: 09/260, concentration 5 × 10^6^ IU/mL), the 1st WHO International Standard for HCMV for Nucleic Acid Amplification Techniques (WHO HCMV standard, NIBSC code: 09/162, concentration 5 × 10^6^ IU/mL), and the RSV Nucleic Acid Detection National Reference Material (code:370057-202001, concentration 3 × 10^8^ U/mL). All clinical specimens were tested in parallel using both the evaluated automated molecular detection system and the routine real-time quantitative PCR (RT-qPCR) platform currently employed in our hospital, which served as the reference standard. Diagnostic performance was assessed based on the results of the reference method.

### Stress test for continuous operational stability

To evaluate the long-term operational stability of the system, a 168-h continuous operation stress test was conducted in accordance with the Technical Guidelines for the Reliability Evaluation of Fully Automated Nucleic Acid Purification and Amplification Detection Systems issued by the National Medical Device Quality Supervision Authority. From April 19 to April 25, 2024, the instrument (PANA HM9000; Serial No.: TL52NL22120001) was continuously powered on for seven consecutive days in the manufacturer’s internal quality control laboratory. During each 24-h cycle, the system completed one full-capacity testing run, processing approximately 2000 samples per day, simulating maximum daily workload. Throughout the testing period, system status, error logs, and output quality were continuously monitored. Key performance indicators—including fault occurrences, interruption events, and error-free run rates—were recorded to assess consistency and robustness under sustained high-throughput conditions.

### Nucleic acid extraction and detection reagents and instrument

The following detection kits were used: EBV DNA detection kit (manufacturer: Suzhou Tianlong Technology, batch number: 20240409), HCMV DNA detection kit (manufacturer: Suzhou Tianlong Technology, batch number: P811240412), and RSV RNA qualitative detection kit (manufacturer: Suzhou Tianlong Technology, batch number: P1982326001). For nucleic acid extraction or purification, the quantitative nucleic acid extraction instrument was paired with the corresponding extraction or purification reagents (manufacturer: Xi’an Tianlong Technology, registration number: Shanxi Medical Device 20210023), and the qualitative nucleic acid extraction instrument was paired with its corresponding extraction or purification reagents (manufacturer: Xi’an Tianlong Technology, registration number: Shaanxi Medical Device 20210030). The PANA HM9000 Automated Molecular Detection Streamline (manufacturer: Xi’an Tianlong Technology; note: the subject of evaluation).

### Concordance rate

The qualitative performance of the detection system was evaluated by calculating the concordance rate between test results generated by the system and those obtained from a reference method using clinically characterized residual samples. This evaluation followed the CLSI EP12 guidelines, which provides a structured framework for assessing binary output examinations, such as positive/negative results, by comparing test outcomes against established reference categorizations (Clinical and Laboratory Standards Institute, 2023; Hiraishi et al., 2021). According to EP12, concordance rate serves as a fundamental indicator of agreement and reliability for qualitative assays, particularly when sensitivity, specificity, and predictive values are not the primary focus. In this study, all qualitative results for EBV, HCMV, and RSV were classified into binary outcomes and compared with results from the hospital’s validated RT-qPCR platform to determine positive, negative, and overall agreement.

### Accuracy

Accuracy evaluation was conducted in accordance with the CLSI guideline EP09, which provides a structured approach for assessing bias through measurement procedure comparison using patient samples (Clinical and Laboratory Standards Institute [CLSI], 2018b). Following this guideline, Using the WHO EBV standard and WHO HCMV standard, EBV DNA and HCMV DNA negative plasma samples were diluted into five concentration gradients for testing (EBV: 5 × 10^5^ IU/mL, 5 × 10^4^ IU/mL, 5 × 10^3^ IU/mL, 5 × 10^2^ IU/mL, 5 × 10 IU/mL; HCMV: 1 × 10^6^ IU/mL, 1 × 10^5^ IU/mL, 1 × 10^4^ IU/mL, 1 × 10^3^ IU/mL, 1 × 10^2^ IU/mL). For each concentration, samples were extracted three times, and each extraction well was tested once. The accuracy was determined by comparing the mean detection values with the theoretical clinical values.

### Linearity

Linearity was assessed according to the CLSI guideline EP06, which provides a structured approach for evaluating whether a quantitative measurement procedure yields results that are directly proportional to the true analyte concentrations across a specified interval (Clinical and Laboratory Standards Institute [CLSI], 2020). Using the WHO EBV standard and WHO HCMV standard, EBV DNA, and HCMV DNA negative plasma samples were diluted into five concentration gradients for testing (EBV/HCMV: 1 × 10^6^ IU/mL, 1 × 10^5^ IU/mL, 1 × 10^4^ IU/mL, 1 × 10^3^ IU/mL, 1 × 10^2^ IU/mL). Each concentration was extracted three times, and each extraction well was tested once. The logarithmic mean of the detected concentrations and the logarithmic values of the dilution ratios were linearly fitted, and the linear correlation coefficient (| r|) was calculated.

### Intra-assay precision

Intra-assay precision was evaluated following the CLSI guideline EP05, which outlines standardized protocols for assessing the repeatability and within-laboratory precision of quantitative measurement procedures (Clinical and Laboratory Standards Institute [CLSI], 2014). According to EP05, precision studies aim to characterize the variability under normal operating conditions across multiple runs and replicates. Using the WHO EBV standard, WHO HCMV standard, and the RSV Nucleic Acid Detection National Reference Material, EBV DNA, HCMV DNA, and RSV RNA negative samples were diluted to three concentration gradients—high, medium, and low (EBV DNA: 2 × 10^4^ IU/mL, 2 × 10^3^ IU/mL, 2 × 10^2^ IU/mL; HCMV DNA: 1 × 10^4^ IU/mL, 1 × 10^3^ IU/mL, 1 × 10^2^ IU/mL; RSV RNA: 1 × 10^4^ copies/mL, 2 × 10^3^ copies/mL, 6 × 10^2^ copies/mL). Each concentration was tested 10 times, and the coefficient of variation (CV,%) was calculated (Plesser, 2018).

### Inter-assay precision

Evaluate Inter-Assay Precision according to CLSI EP05 guidelines (Clinical and Laboratory Standards Institute [CLSI], 2014). Using the WHO EBV standard, WHO HCMV standard, and the RSV Nucleic Acid Detection National Reference Material, EBV DNA, HCMV DNA, and RSV RNA negative plasma samples were diluted to three concentration gradients-high, medium, and low (EBV DNA: 2 × 10^4^ IU/mL, 2 × 10^3^ IU/mL, 2 × 10^2^ IU/mL; HCMV DNA: 1 × 10^4^ IU/mL, 1 × 10^3^ IU/mL, 1 × 10^2^ IU/mL; RSV RNA: 1 × 10^4^ copies/mL, 2 × 10^3^ copies/mL, 6 × 10^2^ copies/mL). Each concentration was tested 5 times per day for 4 consecutive days, and the coefficient of variation (CV,%) was calculated (Goodman et al., 2016).

### Limit of detection

The limit of detection (LoD) was evaluated in accordance with CLSI guideline EP17, which defines LoD as the lowest analyte concentration that can be reliably distinguished from background noise and detected with a 95% probability. Following EP17 recommendations, using the WHO EBV standard, WHO HCMV standard, and the RSV Nucleic Acid Detection National Reference Material, EBV DNA, HCMV DNA, and RSV RNA negative samples were diluted to target concentration levels for detection (EBV DNA: 50 IU/mL, 35 IU/mL, 20 IU/mL, 10 IU/mL, 5 IU/mL; HCMV DNA: 60 IU/mL, 30 IU/mL, 20 IU/mL, 10 IU/mL; RSV RNA: 200 copies/mL, 150 copies/mL, 100 copies/mL). The detection rates at different concentrations were calculated, with the detection rate for the LoD required to be ≥ 95%.

### Interference testing

Interference testing was conducted in accordance with CLSI guideline EP07, which provides a standardized approach to identifying and evaluating the effects of endogenous and exogenous substances that may alter the accuracy of measurement procedures. The goal is to determine whether potential interferents cause significant positive or negative bias in the test results (Clinical and Laboratory Standards Institute [CLSI], 2018a). For the EBV interference testing, weakly positive EBV DNA clinical samples (40 IU/mL) were divided into six groups. Five groups were spiked with the following interfering substances: 0.6 mg/mL bilirubin, 120 μG/mL acyclovir, 150 mg/mL hemoglobin, 60 μG/mL streptomycin, and 60 mg/mL triglycerides. The sixth group was mixed with an equivalent volume of saline as a control. Each sample was extracted three times, and each extraction well was tested once (Hays et al., 2022). For HCMV interference testing, weakly positive HCMV DNA clinical samples (60 IU/mL) were divided into five groups. Four groups were spiked with potential interfering substances: 0.2 mg/mL bilirubin, 10.4 μG/mL ganciclovir, 2.0 mg/mL hemoglobin, and 33 mg/mL triglycerides, respectively. The fifth group was supplemented with an equivalent volume of saline as a control. Each sample was extracted three times, and each well was tested once (Hays et al., 2022). For the RSV interference testing, weakly positive RSV RNA clinical samples (400 copies/mL) were divided into ten groups. Nine groups were spiked with the following interfering substances: 0.45 mg/mL azithromycin, 1% whole blood, 100 μG/mL oxymetazoline hydrochloride nasal spray, 100 μg/mL triamcinolone nasal spray, 16.5 g/L hematin, 18 g/L mucin, 2 mg/mL dexamethasone, 658.5 ng/mL arbidol hydrochloride, and 9 mg/mL sodium chloride. The tenth group was mixed with an equivalent volume of saline as a control. Each sample was extracted three times, and each extraction well was tested once (Hays et al., 2022). For all tests, the difference in Ct values (|*Delta*Ct|) between the experimental groups with interferents and the control group without interferents was required to be ≤ 1.

### Cross-reactivity

Evaluate Cross-Reactivity according to CLSI EP07 guidelines (Clinical and Laboratory Standards Institute [CLSI], 2018a). For the cross-reactivity test, EBV DNA negative clinical samples were divided into seven groups. Six groups were spiked with samples infected with various pathogens (≥ 10^5^ pfu/mL), including Hepatitis B virus (HBV), HCMV, adenovirus (AdV), Influenza A virus (IAV), *Candida albicans*, and *Staphylococcus aureus* (SA). The seventh group was mixed with an equivalent volume of saline as a control. For the cross-reactivity test, HCMV DNA negative clinical samples were divided into six groups. Five groups were spiked with samples infected with various pathogens (≥ 10^5^ pfu/mL), IAV, AdV, EBV, RSV, and *Mycoplasma pneumoniae* (MP). The sixth group was mixed with an equivalent volume of saline as a control. For the cross-reactivity test, RSV RNA negative clinical samples were divided into 12 groups. Eleven groups were spiked with samples infected with various pathogens (≥ 10^5^ pfu/mL), including IAV, Influenza B virus (IBV), AdV, MP, rhinovirus (RV), coronavirus (CoV), parainfluenza virus (PIV), *Streptococcus pneumoniae* (SPN), SA, EBV, and HCMV. The twelfth group was mixed with an equivalent volume of saline as a control. Each sample in all tests was extracted three times, and each extraction well was tested once. The detection result was considered acceptable if all results were negative.

### Carryover contamination detection

Carryover contamination was evaluated following CLSI guideline EP47, which provides a structured framework for assessing reagent carryover effects in clinical measurement procedures. Reagent carryover is defined as the unintended transfer of analytes or reagents between test samples that may cause false-positive results or quantitative bias. EP47 recommends alternating high- and low-analyte concentration samples to simulate worst-case scenarios and evaluate the potential for cross-contamination (Clinical and Laboratory Standards Institute [CLSI], 2024). For EBV and HCMV, high-concentration positive clinical samples (≥ 1 × 10^5^ IU/mL) and negative samples were used. For RSV, high-concentration positive clinical samples (approximately 1 × 10^5^ IU/mL) were used. The samples were processed in alternating sequences of positive and negative samples for extraction. Each sample was extracted once, and each extraction well was tested once, with eight repetitions of the test. The detection result is considered acceptable if all negative samples test negative and all positive samples test positive.

### Statistical analysis

Experimental data were analyzed using SPSS version 27.0 and R version 4.4.1 statistical software. Statistical analysis of the concordance rate was performed using SPSS version 27.0. Descriptive statistics were initially used to summarize the distribution of detection results. Cohen’s Kappa coefficient was calculated to assess the agreement between test kit results and diagnostic outcomes across different groups. Asymptotic standard error and approximate *t*-tests were used to evaluate the statistical significance of the Kappa values. To visually represent the performance metrics of the detection system, we used R packages such as “ggplot2” and “cowplot” to create effective and high-quality visualizations.

## Results

### Stress testing for continuous operational stability

From April 19 to April 25, 2024, the instrument (Model: PANA HM9000; Serial No.: TL52NL22120001) was operated continuously for 7 days, with one full-capacity run (24 h) conducted each day. In each run, the system processed approximately 2,000 samples, simulating its maximum daily workload. All seven runs were completed successfully without any interruptions or error events. No system failures or responsible faults were recorded during the entire testing period, and all sample results met the predefined quality control standards.

### Concordance rate

The detection system was used to test 120 plasma samples and 121 oropharyngeal swab samples. The results showed that the positive concordance rate for EBV DNA, the positive concordance rate was 100% (31/31) and the negative concordance rate was 100% (9/9). For HCMV DNA was 100% (51/51), with a negative concordance rate of 100% (29/29). For RSV RNA, the positive concordance rate was 100% (91/91) and the negative concordance rate was 100% (30/30). The positive concordance rate, negative concordance rate, and overall concordance rate for all three nucleic acid detection kits were 100% (*Kappa* = *1, P* < 0.001).

### Accuracy

**FIGURE 1:**
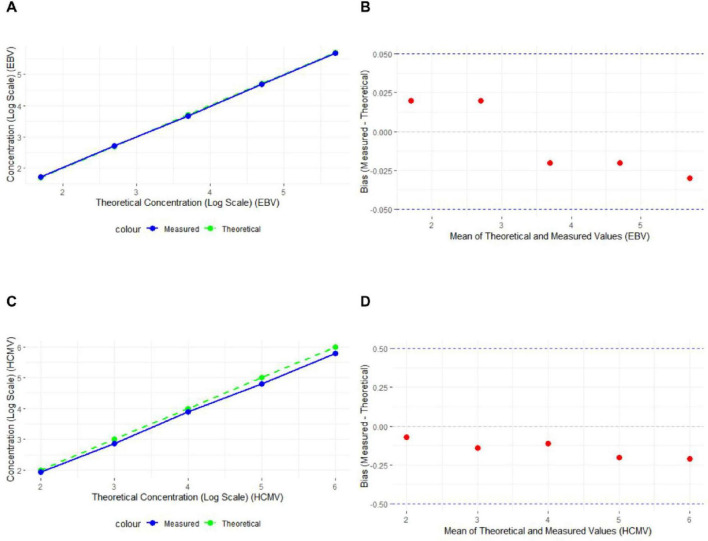
Absolute Logarithmic Deviation of EBV DNA and HCMV DNA Standard Concentrations **(A)** Line graph of EBV DNA experimental results, with the x-axis representing the logarithmic values of the theoretical concentrations and the y-axis representing the logarithmic values of the measured concentrations. The solid blue line represents the actual measured values, and the dashed green line represents the theoretical values (*n* = 15). **(B)** Bland-Altman plot of EBV DNA experimental results, with the y-axis representing the deviation between the measured and theoretical values. The red dots represent the deviation for each sample, and the dashed lines mark the ± 0.05 range (*n* = 15). **(C)** Line graph of HCMV DNA experimental results (*n* = 15). **(D)** Bland-Altman plot of HCMV DNA experimental results (*n* = 15).

The experimental results showed that the absolute logarithmic deviations for the standard concentrations of both EBV DNA and HCMV DNA were within the ± 0.5 log unit range. A strong correlation was observed between the measured and theoretical concentrations for both EBV DNA and HCMV DNA, with the linear regression line closely aligning with the theoretical line (Figure 1).

### Linearity

**FIGURE 2:**
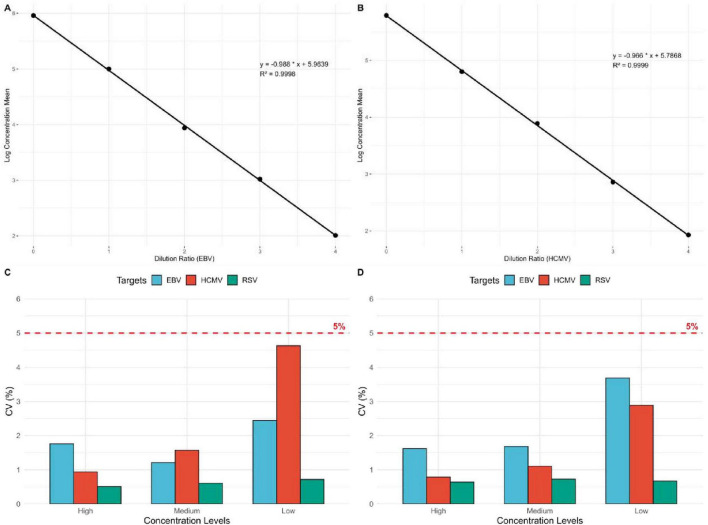
Linear relationship between the logarithmic mean of EBV DNA and HCMV DNA detection concentrations and dilution ratios, and precision of EBV DNA, HCMV DNA, and RSV RNA. **(A)** Linear regression plot of EBV DNA dilution ratios and the logarithmic mean of concentrations (*n* = 15). **(B)** Linear regression plot of HCMV DNA dilution ratios and the logarithmic mean of concentrations (*n* = 15). **(C)** Intra-assay precision evaluation of EBV DNA, HCMV DNA, and RSV RNA. The figure shows the intra-assay precision of EBV DNA, HCMV DNA, and RSV RNA at high, medium, and low concentration gradients. The red dashed line represents the 5% precision standard (*n* = 30, *n* = 30, *n* = 10). **(D)** Inter-assay precision evaluation of EBV DNA, HCMV DNA, and RSV RNA (*n* = 75, *n* = 60, *n* = 60).

The logarithmic values of the diluted standard samples were used, and the average slope method was applied to verify the linearity. The logarithmic means of the measured linear sample concentrations and the logarithmic values of the dilution ratios were linearly fitted, showing a high degree of linear correlation. The linear regression equation for EBV DNA was y = –0.988 x + 5.9639, with a correlation coefficient of *R*^2^ = 0.9998, indicating a quantitative detection linear correlation coefficient |r| ≥ 0.98. The linear regression equation for HCMV DNA was y = –0.966 x + 5.7868, with a correlation coefficient of *R*^2^ = 0.9999, also indicating a quantitative detection linear correlation coefficient |r| ≥ 0.98 (Figures 2A,B).

### Intra-assay precision

The results showed that the intra-assay precision for EBV DNA at high, medium, and low concentrations (2 × 10^4^ IU/mL, 2 × 10^3^ IU/mL, 2 × 10^2^ IU/mL) was 1.76, 1.21, and 2.44%, respectively. For HCMV DNA, the intra-assay precision at high, medium, and low concentrations (1 × 10^4^ IU/mL, 1 × 10^3^ IU/mL, 1 × 10^2^ IU/mL) was 0.94, 1.57, and 4.63%, respectively. For RSV RNA, the intra-assay precision at high, medium, and low concentrations (1 × 10^4^ copies/mL, 2 × 10^3^ copies/mL, 6 × 10^2^ copies/mL) was 0.51, 0.61, and 0.72%, respectively. The coefficient of variation (CV,%) for the logarithmic values of the detected concentrations was ≤ 5% for all samples (Figure 2C).

### Inter-assay precision

The results showed that the inter-assay precision for EBV DNA at high, medium, and low concentrations (2 × 10^4^ IU/mL, 2 × 10^3^ IU/mL, 2 × 10^2^ IU/mL) was 1.62, 1.68, and 3.69%, respectively. For HCMV DNA, the inter-assay precision at high, medium, and low concentrations (1 × 10^4^ IU/mL, 1 × 10^3^ IU/mL, 1 × 10^2^ IU/mL) was 0.79, 1.10, and 2.89%, respectively. For RSV RNA, the inter-assay precision at high, medium, and low concentrations (1 × 10^4^ copies/mL, 2 × 10^3^ copies/mL, 6 × 10^2^ copies/mL) was 0.64, 0.73, and 0.67%, respectively. The coefficient of variation (CV,%) for the logarithmic values of the detected concentrations was ≤ 5% for all samples (Figure 2D).

### Limit of detection

**FIGURE 3:**
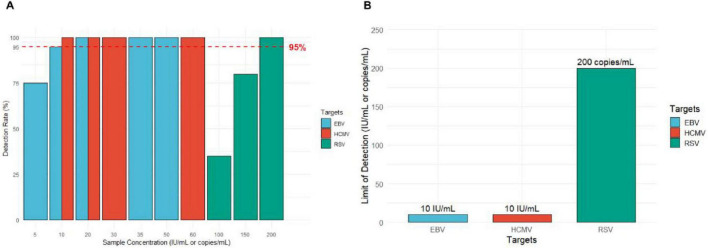
Results of the limit of detection (LoD) experiment. **(A)** Detection rates of EBV, HCMV, and RSV at different nucleic acid concentrations. The red dashed line indicates the 95% detection rate threshold (*n* = 20, *n* = 20, *n* = 20). **(B)** The minimum detection limits for EBV DNA, HCMV DNA, and RSV RNA.

The results showed that for EBV DNA, the detection rates were 100% at concentrations of 50 IU/mL, 35 IU/mL, and 20 IU/mL; 95% at 10 IU/mL; and 75% at 5 IU/mL. The detection rate for the sample concentrations must be ≥ 95%, establishing the LoD for EBV DNA at 10 IU/mL. For RSV RNA, the detection rates were 100, 80, and 35% at concentrations of 200 copies/mL, 150 copies/mL, and 100 copies/mL, respectively. The detection rate for the sample concentrations must be ≥ 95%, establishing the LoD for RSV RNA at 200 copies/mL. The results for HCMV DNA showed a detection rate of 100% at concentrations of 60 IU/mL, 30 IU/mL, 20 IU/mL, and 10 IU/mL. The detection rate for the sample concentrations must be ≥ 95%, establishing the LoD for HCMV DNA at 10 IU/mL (Figure 3).

### Interference testing

**FIGURE 4:**
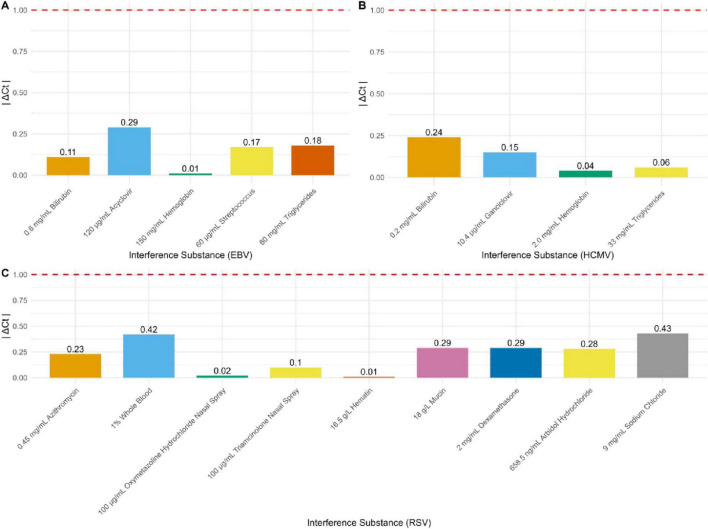
Results of interference resistance for EBV DNA, HCMV DNA, and RSV RNA. **(A)** Interference resistance results for EBV DNA (*n* = 16). **(B)** Interference resistance results for HCMV DNA (*n* = 16). **(C)** Interference resistance results for RSV RNA (*n* = 16).

The detection results for EBV, HCMV, and RSV in the experimental groups with added interfering substances compared to the control groups without interferents showed that |ΔCt| was ≤ 1 for all tests (Figure 4). For EBV, a variety of substances were tested, including 0.6 mg/mL bilirubin, 120 μg/mL acyclovir, 150 mg/mL hemoglobin, 60 μG/mL streptomycin, and 60 mg/mL triglycerides. The |ΔCt| values of 0.11, 0.29, 0.01, 0.17, and 0.18, respectively, all well below the threshold (Figure 4A). Similarly, for HCMV detection, interferents included 0.2 mg/mL bilirubin, 10.4 μg/mL ganciclovir, 2.0 mg/mL hemoglobin, and 33 mg/mL triglycerides. The |ΔCt| values of 0.24, 0.15, 0.04, and 0.06, respectively, all within the acceptable range (Figure 4B). For RSV, the interfering substances included 0.45 mg/mL azithromycin, 1% whole blood, 100 μg/mL oxymetazoline hydrochloride nasal spray, 100 μG/mL triamcinolone nasal spray, 16.5 g/L hematin, 18 g/L mucin, 2 mg/mL dexamethasone, 658.5 ng/mL arbidol hydrochloride, and 9 mg/mL sodium chloride. The |ΔCt| values of 0.23, 0.42, 0.02, 0.1, 0.01, 0.29, 0.29, 0.28, and 0.43, respectively, all well within the acceptable range (Figure 4C).

### Cross-reactivity

The detection results were negative after adding cross-reactive substances to HCMV DNA, RSV RNA, and EBV DNA clinical samples that initially tested negative.

### Carryover contamination rate

Testing was conducted by alternating between positive samples (at specific concentrations) and negative samples. The results showed that positive samples tested positive and negative samples tested negative, demonstrating that no carryover contamination occurred.

## Discussion

With the rapid development of PCR technology, nucleic acid detection has become a core diagnostic tool in medical fields such as infectious diseases and genetic disorders (Zhu et al., 2023). The rapid spread of COVID-19 and monkeypox viruses has further highlighted the importance of fast and accurate detection of infectious pathogens (Harshani et al., 2023; Tang et al., 2020). However, traditional PCR systems have revealed several limitations when faced with the increasing demand for sample testing, including complex operation, a high reliance on specialized technicians, low testing efficiency, and long turnaround times (Kevadiya et al., 2021; Kosai et al., 2022). In response to these challenges, high-throughput automated nucleic acid detection systems have emerged, offering an ideal solution for large-scale pathogen screening by improving testing efficiency and simplifying workflows, thus demonstrating broad clinical application potential.

This study introduces a more robust, standardized, and comprehensive performance validation framework, which was used to conduct both quantitative and qualitative detection of nucleic acids for three pathogens: EBV, HCMV, and RSV. The reference materials were diluted using pathogen-negative plasma samples rather than traditional saline or buffer solutions, effectively avoiding solvent heterogeneity and interference, thereby more accurately simulating clinical testing (FDA Foods Program Regulatory Science Steering Committee [RSSC], 2019). This approach was used to verify multiple performance indicators of the high-throughput automated nucleic acid detection system. The results showed that the system performed exceptionally well in terms of accuracy, precision, interference testing, and linearity. The positive, negative, and overall concordance rates for EBV, HCMV, and RSV RNA detection were all 100%, demonstrating that the system’s performance is comparable to that of existing commercially available detection kits and can reliably reproduce both quantitative and qualitative clinical detection results. Throughout the detection process, the absolute logarithmic deviation of concentrations (the difference between the logarithmic mean of the measured concentrations and the theoretical logarithmic concentrations) for all samples remained within ± 0.5 log units. The logarithmic mean of the measured concentrations showed a high degree of consistency and good fit with the theoretical logarithmic concentrations, indicating that the system has high accuracy. This demonstrates the effectiveness of the system in quantitative pathogen detection, ensuring the reliability of the test results. The system demonstrated excellent precision, with intra-assay and inter-assay coefficients of variation (CV) both ≤ 5%, indicating the nucleic acid detection system has high stability and consistency. The wider the linearity, the stronger the applicability of the system (Pum, 2019a). The results showed that the system had a good linear relationship within the concentration range of [1 × 10^2^ to 1 × 10^6^ IU/mL], with a correlation coefficient |r| ≥ 0.98, ensuring that the system can provide accurate and reliable results across varying pathogen loads. This is of great significance for assessing changes in patient conditions and developing personalized treatment plans (Herdina et al., 2022). The system’s limit of detection (LoD) for EBV and HCMV is 10 IU/mL, which is lower than that of some commercially available nucleic acid detection systems. For instance, the minimum LoD for EBV and HCMV on the cobas 6,800 system is 18.8 and 34.5 IU/mL, respectively (Roh et al., 2021; Lütgehetmann et al., 2023). These detection limits are higher than those of this system, further highlighting the superior sensitivity of this system for detecting EBV and HCMV. This indicates the system’s stability and reliability in detecting low concentrations of EBV and HCMV, ensuring accurate identification of viral nucleic acids even at very low viral loads or during the early and recovery stages of viral infections (Hays et al., 2024). However, for RSV detection, the system’s limit of detection is 200 copies/mL, whereas the Alinity m system developed by Abbott has a limit of detection of 22 copies/mL (Zhen et al., 2022). This indicates that the sensitivity of this system in qualitative RSV detection still has opportunity for improvement. To evaluate whether common endogenous substances and therapeutic drugs would interfere with the system’s results, interference testing was conducted following the CLSI EP07 guidelines, which outline potential interfering substances and the corresponding experimental methods. In the interference tests, common endogenous interfering substances and commonly used therapeutic drugs were added to weakly positive EBV, HCMV, and RSV samples. The system’s detection results showed no significant differences compared to the control group (|ΔCt| < 1), indicating that the system effectively mitigates the influence of interfering substances, thereby avoiding false positives or false negatives. Additionally, to ensure that the system can accurately detect the target nucleic acid in the presence of other common pathogens without being affected by non-target pathogens, cross-reactivity tests were conducted according to the common related pathogens recommended in the CLSI EP07 guidelines. The results of the cross-reactivity tests showed that no non-specific reactions occurred after the addition of non-target pathogens, confirming the high specificity of the system. Finally, in the carryover contamination tests, no false positives were observed when alternating between high-concentration and low-concentration samples, demonstrating the system’s ability to prevent contamination during high-throughput operations.

Compared to traditional manual PCR systems, this automated nucleic acid detection system offers significant advantages. As mentioned earlier, traditional PCR instruments often require multiple manual steps, which increases the risk of laboratory contamination and human error. However, with its automated design, this system achieves a fully automated “sample in, result out” process, greatly reducing the risks of human interference and cross-contamination. To effectively avoid false negatives, the system incorporates internal controls: for EBV and HCMV, the system uses specific primers and probes targeting the conserved viral genes, with probes labeled with FAM fluorescence. Additionally, a synthetic sequence that does not interfere with the target gene is used as an internal standard, with specific primers and probes labeled with Cy5 fluorescence. This ensures consistency in amplification efficiency between the internal control and the target gene, allowing for accurate calculation of viral nucleic acid concentrations based on the internal control. For RSV, the system monitors the entire process from nucleic acid extraction to amplification through normal amplification of GAPDH. The incorporation of internal controls further ensures the accuracy and reliability of the system when detecting complex clinical samples. The system is also compatible with various mainstream blood collection tubes and offers a priority emergency testing pathway, capable of handling eight different tests simultaneously with results available in as fast as 80 min, enabling real-time rapid clinical detection. Furthermore, the system is equipped with a bidirectional connection to the laboratory information system (LIS), allowing for automatic identification of tests and automated result reporting, significantly improving workflow efficiency while avoiding errors associated with manual data entry. This is particularly advantageous in large-scale screening situations. Compared to other automated PCR systems, such as the Roche cobas 6,800 system—which can detect 12 different targets simultaneously and work continuously for 90 days—this system offers a higher sample processing throughput. The Roche cobas 6800 system can process 1,440 samples per 24 h (1440T/24 h), while the system can handle up to 1,776 samples in 24 h (1776T/24 h), greatly reducing sample processing time and significantly enhancing detection efficiency. Huang et al. developed and evaluated a fully automated microfluidic PCR chip system that performed well in terms of precision and contamination control; however, it has certain limitations in high-throughput capability and system runtime (Huang et al., 2021). Similarly, Mirabile et al. (2024) described digital PCR, which offers higher sensitivity and accuracy but still lacks in terms of contamination control, system automation, and high-throughput testing. In contrast, this system adopts a physical partitioning design and a dual isolation door mechanism for contamination prevention, along with HEPA filters, UV disinfection, and gradient negative pressure control to ensure safety during high-throughput operations. Additionally, the system’s five-module independent design enhances its scalability, allowing it to support 1 mL/3 mL extraction systems to meet diverse clinical needs. Its compact size (dimensions: 2,890*1,260*1,750 mm) and high processing speed (1776T/24 h) further enhance its flexibility and efficiency in clinical applications. Furthermore, the successful completion of the 168-h stress test provides additional evidence of the system’s robustness for prolonged high-throughput operations. This type of extended operation is often required in large-scale clinical laboratories and emergency public health situations. The absence of system failures or interruptions over seven consecutive 24-h cycles highlights the platform’s mechanical and software stability, supporting its real-world applicability beyond controlled validation settings. Overall, this system demonstrates superior comprehensive performance.

While this system demonstrates excellent performance overall, several limitations warrant discussion. First, the minimum detection limit for RSV was slightly inferior to that reported for Abbott’s Alinity m platform, indicating room for further optimization in the detection of low viral-load samples. Second, our validation cohort comprised only retrospectively collected plasma and oropharyngeal swab specimens; we did not include prospectively collected or scenario-specific samples such as emergency cases, critically ill patients, pediatric populations, or challenging specimen types (e.g., viscous sputum, bloody fluids). Future work should therefore incorporate real-world, prospectively enrolled samples across diverse clinical contexts to enhance the system’s generalizability. Third, although we evaluated three key viruses (EBV, HCMV, and RSV), routine clinical workflows frequently involve additional matrices: EBV testing may use whole blood and cerebrospinal fluid; HCMV testing commonly includes urine and breast milk; and RSV testing often relies on nasopharyngeal swabs, sputum, or bronchoalveolar lavage fluid (Rzepka et al., 2023; Peuchmaur et al., 2023; Razonable et al., 2020; Onwuchekwa et al., 2023). Large-scale trials with these sample types are needed to confirm the system’s robustness. Finally, while our modular architecture offers flexibility, further hardware and software refinements will be required to support truly multiplexed pathogen panels and accommodate evolving clinical demands. Addressing these points will broaden the system’s applicability in real-world diagnostic settings.

## Conclusion

In conclusion, the high-throughput automated nucleic acid detection system evaluated in this study demonstrated excellent performance in clinical applications, with significant advantages in terms of accuracy, precision, contamination prevention, and efficient detection. Although there is still opportunity for improvement, the system is already capable of handling routine clinical testing tasks and is expected to play a greater role in disease screening, infection monitoring, and public health emergency responses. Additionally, this study summarized a reliable and comprehensive method for evaluating the performance of diagnostic systems, providing valuable insights for future clinical and research applications.

## Data Availability

The raw data supporting the conclusions of this article will be made available by the authors, without undue reservation.
